# Supplementary material for: Transvaginal Ultrasound Accuracy in the Hydrosalpinx Diagnosis: A Systematic Review and Meta-Analysis
Source: Diagnostics (Basel). 2023 Mar 2;13(5):948. doi: 10.3390/diagnostics13050948 (PMC10000875; doi:10.3390/diagnostics13050948)
Supplement: Supplementary file 1 [file diagnostics-13-00948-s001.zip › Supplementary File S1 (search strategy).pdf]

Search in Pubmed

(ultrasound) AND (transvaginal) AND ((hydrosalpinx) OR (adnexal mass))

Filters:

- Language: Eng, FR, Germ, Span
- Years 1990-2022

Citations: 1046

Search in Web of Science

(ultrasound) AND (transvaginal) AND ((hydrosalpinx) OR (adnexal mass))

Filters:

- Language: Eng, FR, Germ, Span
- Years 1990-2022

Citations: 1175

Search in SCOPUS

(ultrasound) AND (transvaginal) AND ((hydrosalpinx) OR (adnexal mass))

Filters:

- Language: Eng, FR, Germ, Span
- Years 1990-2022

Citations: 547

Search in CLINICALTRIALS.GOV

(ultrasound) AND (transvaginal) AND ((hydrosalpinx) OR (adnexal mass))

Citations: 3

Search in Cochrane Library

(ultrasound) AND (transvaginal) AND ((hydrosalpinx) OR (adnexal mass))

Filters:

- Language: Eng, FR, Germ, Span
- Years 1990-2022

Citations: 16
